# An improved breast cancer classification with hybrid chaotic sand cat and Remora Optimization feature selection algorithm

**DOI:** 10.1371/journal.pone.0300622

**Published:** 2024-04-11

**Authors:** Afnan M. Alhassan

**Affiliations:** College of Computing and Information Technology, Shaqra University, Shaqra, Saudi Arabia; University of Manitoba, CANADA

## Abstract

Breast cancer is one of the most often diagnosed cancers in women, and identifying breast cancer histological images is an essential challenge in automated pathology analysis. According to research, the global BrC is around 12% of all cancer cases. Furthermore, around 25% of women suffer from BrC. Consequently, the prediction of BrC depends critically on the quick and precise processing of imaging data. The primary reason deep learning models are used in breast cancer detection is that they can produce findings more quickly and accurately than current machine learning-based techniques. Using a BreakHis dataset, we demonstrated in this work the viability of automatically identifying and classifying BrC. The first stage is pre-processing, which employs an Adaptive Switching Modified Decision Based Unsymmetrical Trimmed Median Filter (ASMDBUTMF) to remove high-density noise. After the image has been pre-processed, it is segmented using the Thresholding Level set approach. Next, we propose a hybrid chaotic sand cat optimization technique, together with the Remora Optimization Algorithm (ROA) for feature selection. The suggested strategy facilitates the acquisition of precise functionality attributes, hence simplifying the detection procedure. Additionally, it aids in resolving problems pertaining to global optimization. Following the selection, the best characteristics proceed to the categorization procedure. A DL classifier called the Conditional Variation Autoencoder is used to discriminate between cancerous and benign tumors while categorizing them. Consequently, a classification accuracy of 99.4%, Precision of 99.2%, Recall of 99.1%, F- score of 99%, Specificity of 99.14%, FDR of 0.54, FNR of 0.001, FPR of 0.002, MCC of 0.98 and NPV of 0.99 were obtained using the proposed approach. Furthermore, compared to other research using the current BreakHis dataset, the results of our research are more desirable.

## 1. Introduction

According to the WHO, 8.8 million individuals worldwide lost their lives to cancer, making it the largest cause of non-accidental deaths worldwide. Worldwide, BrC is a prevalent and deadly illness that affects women. Among many cancers kinds, including brain, liver, and lung cancer, breast cancer ranks third in terms of mortality [1–3]. Furthermore, throughout the next 20 years, there will likely be a 70% rise in the number of new BrC patients. The early and accurate diagnosis is therefore essential to improving the prognosis and raising the survival percentage of individuals. Breast tumors often fall into two types: benign and malignant [4–6]. Malignant tumors are cancerous, whereas benign tumors are non-cancerous. Other subtypes of both tumors require a separate diagnosis since they might result in varying outcomes and treatment strategies [7–9]. Accurate categorization of each BrC category, also known as BrC multi-classification, is necessary for a proper diagnosis.

Compared to other testing methods, clinical imaging techniques are more widely used and very successful in the identification of BrC [10, 11]. In real-world scenarios, pathologist spends a great deal of time analyzing the pathological image and significantly depend on their clinical expertise, which increases the risk of misclassification [12–14]. To qualitatively analyze histopathology images, it is evident that sophisticated and automated image processing techniques are required. With the development of computer vision, computer-aided diagnosis (CAD) is utilized to accelerate diagnosis and reduce misdiagnosis rates [15–17]. One kind of CAD approach is deep learning. It uses multiple-layered artificial neural networks to learn and extract characteristics from data [18–20]. DL-based breast cancer histopathology image categorization outperforms the conventional approach with a considerable performance gain and continues to advance in this sector.

Thus, this work aims to further assess the efficacy of the feature extraction approach using DL for BrC diagnosis and classification. The ASMDBUTMF is first used to preprocess the input image. Next, image segmentation is carried out using the technique that is set to the Threshold Based Level. After segmentation, a novel hybrid chaotic sand cat optimization and a feature selection approach based on the Remora Optimization Algorithm are implemented. The proposed method facilitates the acquisition of precise functionality attributes, hence simplifying the detection procedure. Finally, a DL classifier called CVA is employed to distinguish between benign and malignant tumors. To guarantee the DL-BrC model’s efficient diagnostic performance, a large number of simulations are run. The following is our main contribution to this research.

- The proposed framework is divided into many stages: segmentation, selecting features, categorization, and preprocessing. Accurate identification and classification of breast cancers is ensured by employing an exhaustive strategy.
- To put in place a productive pre-processing technique using the ASMDBUTMF, medical histopathology breast images can be free of high-density noise.
- To employ a hybrid FS technique. The drawbacks of traditional methods are addressed by the inclusion of the hybrid feature selection technique known as chaotic sand cat optimization with the Remora optimization algorithm. High computational effectiveness, scalability, and the capacity to select the most pertinent and instructive characteristics for precise detection are all provided by this method.
- To accurately distinguish between benign and malignant tumors by implementing a Conditional Variation Autoencoder model for categorization. It enhances our system’s performance.

The following is the format of the upcoming parts. Section 2 discusses current studies on the detection of BrC using various learning approaches. In Section 3, the method recommended for the research study is presented. In Section 4, the result is formalized and discussed. The study is concluded in Section 5 with recommendations for further research.

A thorough explanation of the existing approaches has been given in the literature survey section. The overview of earlier research that examined various learning strategies for predicting the beginning of breast cancer is provided below:

In 2021, *Karimi Jafarbigloo et al*. [21] introduced two automated algorithms leveraging deep learning techniques for grading nuclear atypia in breast cancer histopathology images. Using a patch-based methodology, System I identified important patches in the photos. Convolutional neural networks (CNNs) with three hidden layers were created and trained for patch categorization and feature extraction. In System II, all patches were taken into consideration concurrently by a two-layer Long Short-Term Memory (LSTM) network for image classification and CNN for feature extraction. After normalizing the input photos, training and test datasets were separated during the preparation stage. The photos in the training dataset were further divided into patches, and the corresponding image grade was assigned to each patch. These patches were then used as inputs for the feature extraction and classification methods that were suggested.

An attention high-order deep network (AHoNet) was introduced in 2021 by *Zou et al*. [22] to classify images related to breast cancer histology. The study concentrated on using DL techniques for computer-aided categorization of pathological images related to breast cancer. A channel attention module was used by AHoNet to retrieve pertinent information from diseased images. It calculated second-order covariance statistics using matrix power normalization to offer another trustworthy global characteristic representation. The model incorporated an attention strategy and high-order statistical visualization into the preexisting convolutional network. Using two publicly available breast cancer pathology datasets, BreakHis and BACH, the authors thoroughly assessed AHoNet.

The FE-Bk CapsNet method for the automated classification of breast cancer histopathology images was introduced by *Wang et al*. [23] in 2021. To produce more discriminative features, the technique combines the benefits of a CapsNet and a convolutional neural network (CNN). To extract convolution features and capsule features simultaneously, combining semantic and spatial features into new capsules, a special structure with dual channels was created. By altering the loss function and integrating the routing procedure into the overall optimization process, the routing coefficients were adaptively and indirectly optimized. The effectiveness of this strategy for classifying breast cancer in clinical settings was proved by the experimental results, which were conducted using the BreaKHis public database. The study emphasized the value of early breast cancer detection as well as the drawbacks of traditional handcrafted feature-based methods.

*Ahmad et al*. [24] presented a transfer learning-based method in 2021 for classifying histopathology images to assist in the diagnosis of breast cancer. Because of its capacity for automatic feature extraction, the article emphasized a shift away from traditional ML methods and toward DL models, namely CNN. Deep learning-based methods were created to tackle disagreements between pathologists and other healthcare professionals to increase decision consistency, boost productivity, and lower mistakes in the medical field. The authors presented a method for identifying histological images for the detection of breast cancer that is based on deep learning and transfer learning. Whole slide images were used to extract patches, which were then fed into a CNN to extract features. A pre-trained Efficient-Net architecture on the ImageNet dataset was fed discriminative patches. Additionally, an SVM classifier was trained using features that were taken from the Efficient-Net architecture.

*Boumaraf et al*. [25] in (2021) presented a transfer learning-based method for the automated categorization of breast cancer from histopathological images. The method includes binary and eight-class categorization that account for both magnification-dependent (MD) and magnification-independent (MI) features. The task was given to the DNN ResNet-18, which had been previously trained on ImageNet. To reduce overfitting and speed up training, a block-wise fine-tuning technique was used to make the final two residual blocks of the network more domain-dependent to the target data. To improve the approach’s versatility, three-fold data augmentation on training data and global contrast normalization (GCN) depending on the target’s data values were applied. The efficacy of this strategy was proved by the experimental results on the BreaKHis dataset. To complete the MD classification task, GCN was used independently for every four magnification factor datasets (40x, 100x, 200x, and 400x).

In 2021, *Hao et al*. [26] developed a low-dimensional three-channel feature-based technique for the identification of breast cancer pathology images. Ten descriptors, including GLCM1, GLCM4, APVEC, HIM, wavelet features, Tamura, CLBP, LBP, Gabor, and Hog, were extracted. The performance of these characteristics was evaluated using SVM. The article also addressed the improvement of recognition accuracy through the fusion of multiple characteristics in a cascade manner. It was evaluated under the BreaKHis dataset.

To classify histopathology images of BrC, *Kashyap et al*. [27] introduced a DL-based classification model in 2022 called the SDRG. The SDRG model used several techniques to address challenges and improve accuracy, including data augmentation with multiple factors to address overfitting; a suggested multiscale stochastic and dilation unit to extract and enhance low-level data, and a ghost unit to remove unnecessary or equivalent data from the convolution neural network.

To detect discriminative areas in HPIs and remove imaging variables, *Demir et al*. [28] presented a pre-processing phase in 2021 utilizing the MWSA. The processed HPIs were used to train a CLSTM model, which comprised an LSTM model for classification and an SDC model to transform HPIs into 1D data. It transmits neuron values from the CLSTM model to an optimized SVM for classification.

*Chaudhury et al*.*’s 2021* research [29] concentrated on the categorization of BrC histopathology images through the implementation of a unique method known as bilateral knowledge distillation and LSR. The authors emphasized the value of breast cancer prevention and research, as well as the ability of data mining techniques to extract relevant information from large, complicated databases. With a network of greater capacity functioning as an instructor and a network of lesser capacity functioning as a learner, they established the general idea of knowledge distillation. Additionally, they presented a brand-new technique for BKD that allowed for several interactions between student and instructor models, all of which improved the performance of the other.

The FabNet model, a Features Agglomeration-Based CNN for Multiscale BC Histopathology image categorization, was introduced by *Amin et al*. [30] in 2023. Through the use of an agglomerative architecture that acquired layers close to merge semantic and spatial details for cancer image categorization, the model was created to learn the fine-to-coarse structural and textural aspects of multi-scale histopathological images. Two widely accessible standard datasets of colorectal and breast cancer are used by the FabNet model. The suggested model was trained with the retrieved pictures to identify and distinguish between benign and malignant tumors. The method used for the study involved preprocessing the dataset using stain normalization and extraction of patches methods to acquire training examples.

A ground-breaking work by *Singh et al*. [31] concentrated on creating a sophisticated predictive model for the early-stage identification of breast cancer. The authors proposed a novel method based on Eagle Strategy Optimization (ESO), Gravitational Search Optimization (GSO) algorithm, and their hybrid to address the complexity of feature selection, a crucial issue in machine learning and massive data preprocessing. Using the Wisconsin Diagnostic Breast Cancer (WDBC) dataset, the goal was to classify breast cancer using the greatest accuracy and the fewest features possible. The researchers demonstrated the efficacy of their implemented methods, which had not been employed for this topic before, by creating a predictive model that categorized breast cancer tumors by combining soft computing technologies and machine learning algorithms. Rigorous experimentation, including 12 experiments with cross-validation and the split approach, validated the proposed approach. The hybrid ESGSA feature selection strategy demonstrated its merits by improving classification accuracy, enhancing the informative quality of features, and reducing processing costs.

Feature selection was initially described by *Singh et al*. [32] as an essential data pre-processing technique for optimizing machine learning (ML) models, with an emphasis on recently popular metaheuristic algorithms. Three metaheuristic feature selection techniques were introduced in the paper: the Emperor Penguin Optimization (EPO) algorithm, the Gravitational Search Optimization Algorithm (GSOA), and an integrated algorithm (hGSEPO) that combines the EPO and GSOA. Notably, the hGSEPO strategy reduced complexity by combining a more exhaustive local search by EPO with a global search by GSOA to find important traits and discard unnecessary ones. This innovative hybrid approach addressed the crucial feature selection step in huge datasets, especially for predicting medical disorders like breast cancer (BC), and was used for the first time in the classification of BC. The study utilized the benchmark BC Wisconsin Diagnostic Breast Cancer (WDBC) feature set, aiming to classify data into two classes while determining the minimum features required for enhanced accuracy.

*Singh et al*. [33] emphasized the pivotal role of feature selection (FS) in reshaping high-dimensional data for machine learning (ML) efficiency. Their study introduced the GWO and WOA, combined in a hybrid version (hGWWO), and applied to the ORIGA dataset. The FS approach, focusing on influential traits, enhanced ML classifiers’ efficiency by reducing computational complexity. Motivated by global glaucoma prevalence and challenges in diagnosis, the authors proposed an AI-supported clinical decision system (CA-CDSS) addressing labor-intensive manual screening. For feature selection and machine learning categorization, the system made use of computers inspired by nature.

In their comprehensive study of the fields of data mining and machine learning (ML), *Singh et al*. [34] emphasized the significance of feature selection (FS) as a vital data preprocessing method for enhancing ML model performance. The authors presented a trio of novel metaheuristic feature selection strategies: the Emperor Penguin Optimization Algorithm (EPO), the Bacterial Foraging Optimization Algorithm (BFOA), and a hybrid strategy called hBFEPO that combines the two. Although these algorithms had already been used for several machine learning tasks, their new application in the categorization of breast cancer was a noteworthy advance. First, the COVID-19 dataset was used for preliminary testing, which yielded encouraging findings. Next, the WDBC Breast Cancer dataset was evaluated. The study highlighted how crucial effective feature selection is for minimizing computational expenses and improving categorization results, especially when it comes to breast cancer prediction. The authors pushed for the use of machine learning and computing methods inspired by nature in the creation of clinical decision support systems for improved BC identification.

For the categorization of glaucoma, *Singh et al*. [35] developed a hybrid feature selection strategy based on the Emperor Penguin Optimization algorithm and the Bacterial Foraging Optimization algorithm. To enhance performance across a range of efficiency criteria, the study sought to apply a feature set for categorizing images into two groups (infected and normal). 36 features in all were taken out of the retinal fundus benchmark images. The hybrid feature selection approach increased proficiency in classification while reducing the number of characteristics. Six machine learning classifiers were categorized using these optimization strategies, using less number of features. Eight performance metrics with a statistical foundation were also computed. The study’s maximum accuracy was attained by combining the random forest classifier with the hybrid optimization technique.

The limitations discerned from the literature collectively highlight the challenges in the realm of feature selection for BrC categorization using histopathological images. Key constraints encompass the paucity of labeled images, restraining the optimal exploitation of Convolutional Neural Networks (CNNs). Additionally, conventional feature extraction methods like SIFT and GLCM exhibit shortcomings in capturing the nuanced characteristics of breast cancer histopathological images. The intrinsic variability within and between classes poses a substantial hurdle for precise classification, especially in the context of multiple classes. The dissociation between magnification-dependent and magnification-independent classification methodologies impairs the effectiveness of the FS approach. The intricate nature of previous models further complicates the interpretation and practical application of selected features. Moreover, the demand for substantial data and computational resources, coupled with the potential risks of overfitting and underfitting, presents challenges specific to feature selection. Mitigating these limitations necessitates the development of more robust, interpretable, and generalizable feature selection models. In this work, strides are made to address and overcome these limitations through innovative approaches to feature selection in breast cancer histopathological images.

## 2. Methods

BrC is the most prevalent and quickly spreading disease. Early diagnosis is essential in preventing breast cancer. With early case discovery, many cases may be managed and the death rate can be brought down. Four steps are included in this work’s technique for predicting and categorizing breast cancer: pre-processing of the images, segmentation of the breast, FS, and categorization. The median filter is used to remove unwanted noise from an image and to increase contrast in it. Following the pre-processing stage, level set segmentation is implemented and the breast is distinguished from the breast image using a thresholding technique. The segmented input will be used in the characteristic selection process once the images have been divided into pieces.

**Fig 1:**
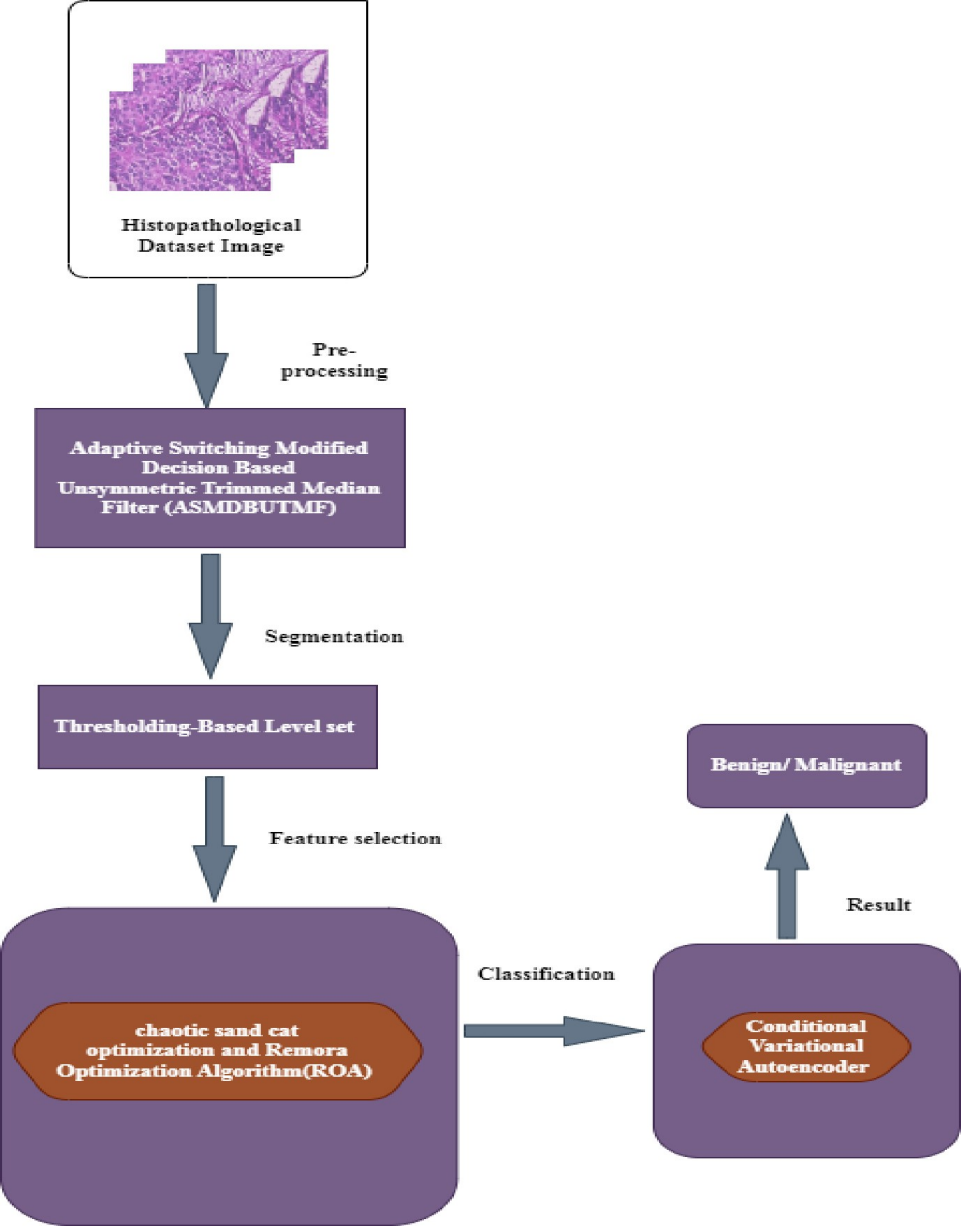
Proposed methodology.

One unique approach to solving optimization issues is the combination of the ROA [36, 37], a hybrid technique, and CSCO in feature selection. The proposed strategy facilitates the acquisition of precise functionality attributes, hence simplifying the detection procedure. It also aids in resolving problems pertaining to global optimization. The output of the feature selection step is sent to the classification procedure. For the categorization of anomalies associated with breast normality, it is advised to use a DL classifier built on a CVA. The proposed approach is extremely effective in increasing classification precision. Fig 1 displays the proposed model architecture diagram.

### 2.1 Dataset collection

**Fig 2:**
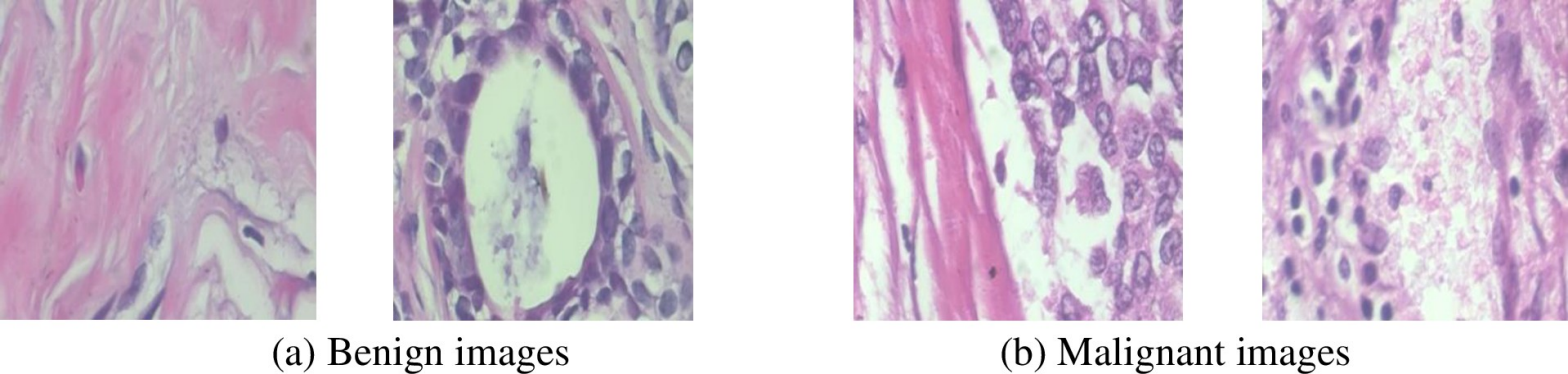
Sample images from the BreakHis dataset. (a) Benign images, (b) Malignant images.

The first large-scale, publicly available dataset of breast cancer histological images that are not full-field is called BreakHis [38, 39]. It is made up of 7909 clinical breast tumor histological images from 82 people, 2480 of which are benign tumors and 5429 of which are malignant tumors. These images are used to confirm the validity and broaden the scope of the proposed model. For each of the three processes, we used data augmentation to solve the problem of imbalanced datasets. We first turned them 90 degrees, and then we mirrored them horizontally. Sample images from the BreakHis dataset are displayed in Fig 2.

### 2.2 Pre-processing

High-density noise is eliminated from medical HPIs using the proposed ASMDBUTMF technique. The present research offers an approach to removing high-density noise from an input. A 3 × 3 size window’s pixels are all made up of noisy pixels. The subsequent components comprise this algorithm.

- NPs identification
- Noise elimination when whole neighbors are NPs
- Noise elimination when NNPs are available

High-density noise is eliminated by using the pixels with values of 0 or 255, which are identified as noisy pixels. The precise elimination of sounds from the clinical images is the focus of the next two circumstances, or instances.

#### 2.2.1 NP identification

The presence of high-density distortion in pathology samples yields 0s and 255 for NPs associated with pepper and salt distortion classes. The input H is illustrated as a set K using Eq (1).

K={0,i,255} (1)

Where, *i* € [1,254]

The aberrant regions *Np*^*ij*^ are recognized based on Eq (2)

$$NH^{ij}=\left\{\begin{array}{c} 1ifH^{ij}==0\\ 1ifH^{ij}==P\\ 0ifH^{ij}>=1\&\&H^{ij}<=P-1 \end{array}\right\}$$ (2)

By employing a single step for noise identification and two steps for noise elimination, our approach improves NP reduction. For the next image processing operations, the noise-free image is utilized.

#### 2.2.2 Noise elimination when whole neighbors are NPs

In a 3 × 3 window, the surrounding pixels around the Processing pixel *H*^*ij*^ are all noisy or a mix of noisy and non-noisy pixels. This section deals with the situation where all of the neighbors are making noise to obtain an image without distortion. Using a 3 × 3 size patch centered on *H*^*ij*^, the neighbor elements are gathered by Eq (3).


$$W_{3\times 3}^{m+1,n+1}=H_{i+m,j+n}$$ (3)

$$\text{m}\;€[-\text{fix}(\frac{\text{s}}{2})|,\;\text{fix}(\frac{\text{s}}{2})]$$

$$n€[-\text{fix}(\frac{\text{s}}{2})|,\;\text{fix}(\frac{\text{s}}{2})]$$

In this case, m stands for the row indication index, and W_3X3_ indicates the 3 × 3 size patch. The terms "n" and "s" denote the index and window size, respectively (considering 3) Using Eq (4), the erroneous segments count C_NP_ is calculated from the s×s window.


$$C_{NP}=0$$

$$C_{NP}=\left\{\begin{array}{c} C_{NP}=C_{NP+1,ifW_{3\times 3}^{m,n}==0||W_{3\times 3}^{m,n}==255}\\ C_{NP,else} \end{array}\right\}$$ (4)

C_NP_—Noisy byte count is where It may be determined that all of the pixels that are noisy in the window W_3X3_ are present if the noise pixel count is C_NP_ = 9. We choose a 5 × 5 frame and look for pixels that are not noisy. The provided 5 × 5 window components will be used to reduce noise if NNPs are available. A 7 × 7 pixels chosen and its non-noisy pixel accessibility will be evaluated if NNPs are not accessible. The 7 × 7 window will be used to perform noise mitigation if without distortion pixels are presented. If this is not the case, a 9 × 9 window will be chosen and the presence of NNPs will be verified.

#### 2.2.3 Noise elimination while NNPs are available

Two various sizes of overlapping windows, such as 3 × 3 and 5 × 5, are used to resolve or restore the present noisy pixel that is surrounded by the smallest amount of non-noisy pixels as a noise-free pixel. The CNNP is computed and a 3 × 3 size window is chosen. The median value takes the place of the current noisy pixel if the CNNP falls between 3 and 8. If this criterion is not met, the 0^th^ component of the sequentially stored NNPs of the 3 × 3 size window is allocated to the α value, and the first element of the same is assigned to the β value. After that, a 5 × 5 window is taken out, and CNNP is calculated. If CNNP > 3, the noisy pixel is swapping out with the average of the 5×5 windows. If CNNP equals 2, the mean of α and β is used to swap out the noisy pixel that is now present. In the noise-free image, the α value is preserved rather than the noisy value if there is just one NNP surrounded by the NP.

### 2.3 Dataset augmentation

Data augmentation was employed by researchers to improve deep learning techniques. The medical sector lacks sufficient training data sets for DL models, which need more information. Augmenting data is needed to improve the diversity of the source datasets.

### 2.4 Integration of thresholding-based level set segmentation

Level-set segmentation based on thresholding is used for pathology image segmentation; the following sections explain each methodology: The integration of thresholding is a method for selecting a threshold based on a few visual characteristics. If the threshold value of the method is different from the discrete images, it selects a pixel. Moreover, one common technique for figuring out the threshold value is to examine the plots of the images. A weight update unit is used to establish the proper threshold value for bimodal images in the integration of thresholding. The terms μ_1_ and μ_2_ indicate the starting weights that were applied, if we regard the image’s size as [M * N]. The pixel values in the [M * N] image is then contrasted using these weights. The nearest weight is used to update the weight of each input pixel. The difference between the closest weight and the input pixel is added to the closest weight after being multiplied by the learning rate. The word " μ_1_" is updated as it gets nearer to that pixel value, but the term " μ_2_" is updated when it gets even closer. This is represented mathematically using the formula found in Eq (5).


$$\mu_{new}=\;\mu_{old}+\;\beta *\left(pixel-\mu_{old}\right)$$ (5)

The learning rate is represented by Eq (2), where the weight is supplied by and $\beta =\frac{256-px}{256}$. After exposing the updated weights to further image pixels, the threshold value is determined by averaging these two weights. This is presented in Eq (6).


$$\;In^{AT}=\frac{\mu_{1}+\;\mu_{2}}{2}$$ (6)

The image is converted to binary format by adding a threshold value. It is easy to identify an object since pixels with values above *In*^*AT*^ are classed as objects, while those with values below *In*^*AT*^ are categorized as background. Image segmentation can benefit from level-set segmentation. Representing fields or slopes as a zero-level set of a higher-dimensional hyper-surface is the fundamental assumption. Simple topological changes and precise numerical results are possible using the level set approach. The smoothing function of the surface *φ*(*a*, *b*, *t*), is defined as follows, whereas the definitions of the curves are expressed as *φ*(*a*, *b*, *t*) = 0. Thus, the evolution of the curve yields the construction of a 3D-level set function. Examine a level set function represented by *φ*(*a*, *b*, *t*) = 0. The curve acts as the border, dividing the surface into internal and exterior curve segments. The SDF is given on the surface by Eq (7). The shortest path between an area on the surface and its *x*-coordinate is represented.


$$\varphi \left(a,\;b,\;t\right)=\;0=sd$$ (7)

The points of the curve are suitable for the computation indicated in Eq (8) during the development phase. The standard level set motion equation is also indicated by Eq (9) where SF stands for the speed function. The function that creates surface and image attributes is referred to as SF. The total energy function is minimized by the gradient flow resulting from the level set function, and the corresponding energy function of the level set is given.


$$\varphi \left(a,\;b,\;t\right)=\;0$$ (8)

$$\varphi_{t}SF\left|\Delta \varphi \right|=0$$ (9)

$$Eng\;\left(\varphi \right)=\;\mu Int\left(\varphi \right)+\varepsilon ed\lambda ,0\left(\varphi \right)=\;\mu \int_{Ω}^{0}\frac{1}{2}{\left(\left|\Delta \varphi \right|-1\right)}^{2}\text{dxdy}+\lambda \int_{Ω}^{0}edW\left(\varphi \right)\text{dxdy}$$ (10)

The parameter that penalizes the discrepancy between φ and SDF is represented by the notation *μ* > 0. Eq (11) defines the function *ed*.


$$ed=\;\frac{1}{1+\left|\Delta GS_{\sigma }*In^{AT^{2}}\right|}$$ (11)

In the following equation, *Int*(*φ*) and ε(φ) represent the internal and outside energy components, respectively. The *ed* operation, which penalizes departures from SDF using a value μ > 0, is provided by Eq (11).

### 2.5 Feature selection using hybrid algorithm (CSCO-ROA)

The subsequent sections delineate the many methodologies employed in the process of selecting image features through the utilization of CSCO-ROA, a unique contribution to feature selection—a hybrid algorithm designed to address global optimization challenges. The algorithm’s local and global search abilities are significantly enhanced by aggressive behavior. Details on the CSCO-ROA algorithm is given in the section that follows.

#### 2.5.1 Chaotic sand cat optimization algorithm (CSCO)

The ability of sand cats to recognize low-frequency noises in their surroundings is a distinctive property that gives rise to the name of the sand cat optimization [40, 41] approach. The two main activities of the sand cat are feeding on its prey and attacking it. The sand cat has remarkable frequency absorption for wavelengths under 2 kHz, based on research by scientists. These special characteristics enable the sand cat to track prey, detect acoustic signals of movement in the prey, and effectively hunt by using the location of the prey.

In the SCO algorithm, every sand cat stands for a different issue variable. To begin the SCO approach, a random generator generates a potential matrix of the sand cat colony beginning with the smaller and higher bounds of the intended factors. For a d-dimensional optimization area with n sand cats, the size of the candidate matrix is equal to *K*_*pop*_ × *K*_*d*_, (*pop* = 1,…‥,*k*).

Additionally, each sand cat’s fitness value, or cost, is determined using a particular fitness function. Every potential solution, or sand cat, will yield a value for the associated function. To depict this process and facilitate the mathematical modeling of the method, the vector $\vec{r_{G}}$ denoted by the following equation is established. It decreases linearly from two to zero as the number of iterations increases:

$$\vec{r_{G}}=S_{M}-(\frac{S_{M}\times t}{t_{Max}})$$ (12)

Given that the *S*_*M*_ value depends on the hearing features of sand cats, it should be 2.


$$\vec{Pos}(t+1)=\vec{r}.(\vec{Pos_{b}}(t)-rand(0,1)\cdot \vec{Pos_{c}(t)})$$ (13)

Each sand cat has the sensibility range that it uses to escape the local ideal trap ($\vec{r}$), which, utilizing Eq (14), is obtained.


$$\vec{r}=\vec{r_{G}}\times rand(0,1)$$ (14)

This $\vec{r_{G}}$ represents the standard sensibility value, which is decreased from 2 to 0. Furthermore, $\vec{r}$ presents the sensibility value of the individual. Once located, each sand cat’s location is updated in a subsequent SCO phase based on the following equation:

$$\vec{Pos}(d+1)=\vec{Pos_{b}(d)}-\vec{r}\cdot \vec{Pos_{rnd}}\cdot \text{cos}(\theta )$$ (15)

Where *θ* is a random dimension between 0 and 360 and $\vec{Pos_{rnd}}$ regulates the location of a randomly chosen sand cat depends on the subsequent formula:

$$\vec{Pos_{rnd}}=|rand(0,1)\cdot \vec{Pos_{b}}(d)-\vec{Pos_{c}(d)}|$$ (16)

$$\vec{R}=2\times \vec{r_{G}}\times rand(0,1)-\vec{r_{G}}$$ (17)

The search agents are pushed to exploit by the SCO algorithm when R is equal to or less than 1, but they are encouraged to explore and find prey. As a result, Eq (18) is the SCO algorithm’s final modifying position equation.

X→(d+1)=Pos→b(d)−Pos→rnd(d)⋅cos(θ)⋅r→|R|≤1;exploitationr→⋅(Pos→(d)−rand(0,1)⋅Pos(d)c→)|R|>1;exploitation (18)

Eq (18) states that when |*R*| ≤ 1, Sand cats are trained to strike their target; if not, they are tasked with searching the global region for a new, workable solution. The objective of the research is to implement the global search capacity of the SCO. To do this and increase the algorithm’s exploring capability, the chaotic sequence is used in the threshold variable of Eq (14). Deterministic systems having randomness, irregularity, and stochastic characteristics that are impacted by the starting conditions are known as chaotic events. Because of their intrinsic irregularity, chaotic parameters can vary throughout a range of values without rephrasing. A chaotic value may produce chaotic movement and exhibit some kind of chaotic structure. In this research, a popular logistic map is employed in conjunction with the following equation:

μ(d+1)=a×μ(d)×(1−μ(d)) (19)

The chaotic map is indicated by *μ*(*d*), the number of iterations is indicated by t, which stands for a constant equal to 4, and μ (0) should not equal 0, 0.25, 0.5, 0.75, or 1. In the CSCO, the ($\vec{r}$) evaluation equation for the sensitivity range (i.e., Eq (14) uses the chaotic map μ rather than a simple random number to improve the technique’s stochastic behavior and avoid premature convergence. Consequently, the following equation will be used to update a sand cat’s position:

$$\vec{X}(d+1)=\left\{\begin{array}{c} \vec{Pos_{b}}(d)-\vec{Pos_{md}}(d)\cdot \text{cos}(\theta )\cdot \vec{r_{G}}\times \mu \;\;\;\;\;\;\;\;\;\;\;|R|\leq 1;\;\text{exp}\;loitation\\ \vec{r_{G}}\times \mu \cdot (\vec{Pos_{bc}}(d)-rand(0,1)\cdot \vec{Pos_{c}}(d)) \end{array}\right.$$ (20)

Additionally, the weakest sand cat that offers the greatest degree of fitness (in minimization issues) will be replaced with a new one at each of the iterations in the proposed CSCO to improve the algorithm’s exploration and search capabilities. This is illustrated in the following equation:

$$x_{worst}=\left\{\begin{array}{cc} rand_{1}\times \vec{Pos_{b}}(d) & ifrand_{3}\leq 0.5\\ x_{i\;\text{min}}+rand_{2}\times (x_{i\;\text{max}}-x_{i\text{min}}) & ifrand_{3}>0.5 \end{array}\right.$$ (21)

**Fig 3:**
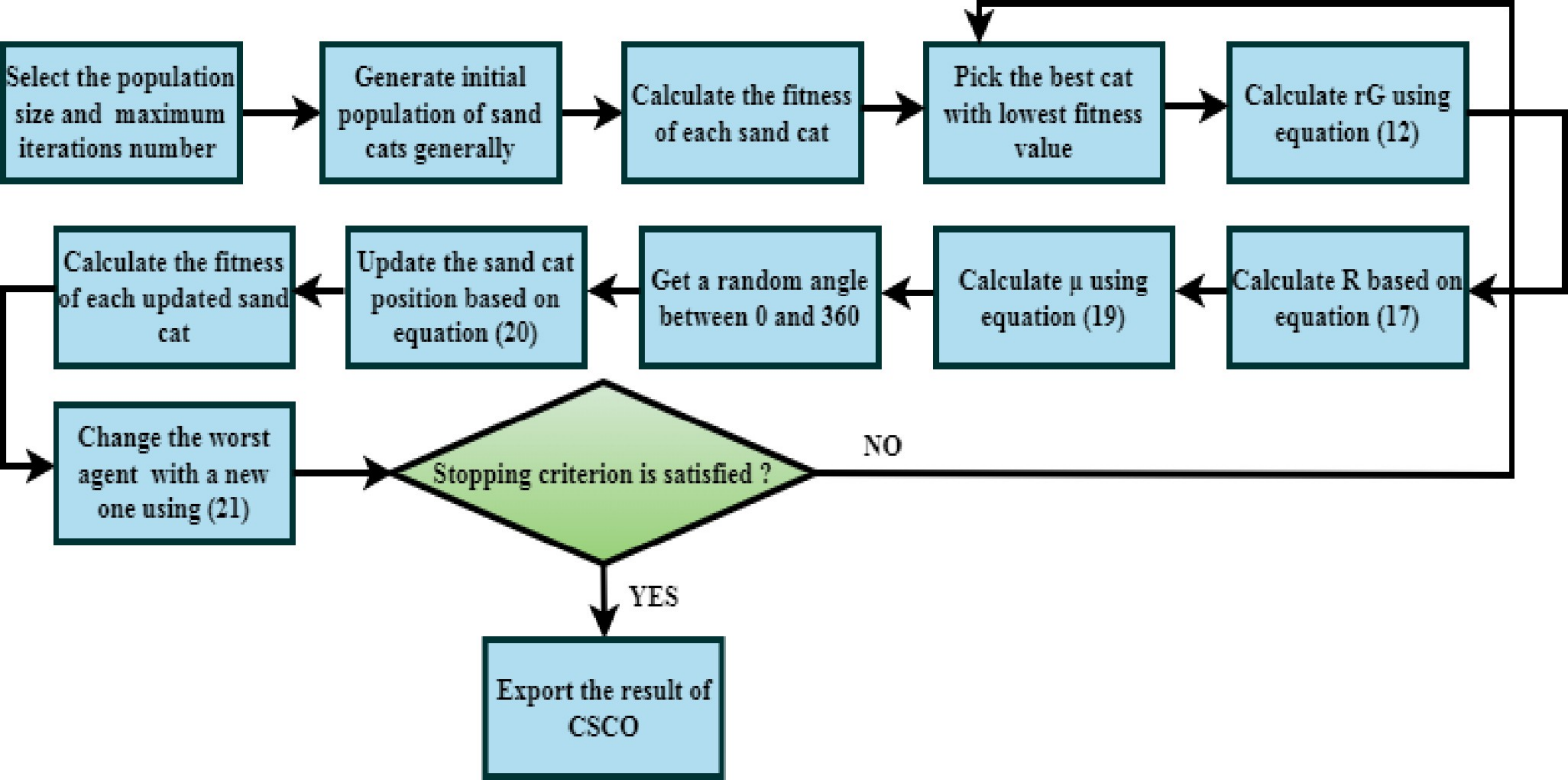
CSCO’s flowchart.

Where *rand*_1_,*rand*_2_, and *rand*_3_ are random numbers between 0 and 1 and *x*_*worst*_ is the sand cat with the greatest fitness rating. The process of CSCO is shown in Fig 3.

#### 2.5.2 Remora Optimization Algorithm (ROA)

Based on the symbiotic link between remoras and sharks in the marine ecosystem, the ROA is an optimization method inspired by nature. With the use of a unique sucker disc, remora fish cling to larger aquatic animals like sharks. The original ROA and its steps are presented in this section.

*2*.*5*.*2*.*1 Free travel (Exploration)*. ***2*.*5*.*2*.*1*.*1 SFO strategy*.** The supreme idea produced by Eq (22) served as the basis for modeling the formulation of this algorithm’s location update.

$$R_{i}^{t+1}=R_{best}^{t}-(rand\times (\frac{R_{best}^{t}-R_{rand}^{t}}{2})-R_{rand}^{t})$$ (22)

Where, $R_{rand}^{t}$ is a unique place.

***2*.*5*.*2*.*1*.*2 Experience attack*.** Similar to the acquisition of information, the tuyu must periodically make small movements around the host to determine whether or not the replacement of the host is necessary. The following formula may be used to describe the aforementioned principles:

$$R_{att}=R_{i}^{t}-(R_{i}^{t}-R_{pre})\times randn$$ (23)

Where, *R*_*pre*_ is the angle of the earlier versions, and *R*_*att*_ is a temporary step.

This step’s decision is defined as a comparison of the fitness functions of the current solution, f ($R_{i}^{t}$), and the proposed solution, f (*R*_*att*_). For instance, in solving the minimum issue, if the proposed solution’s fitness parameter value is lower than the current one,

$$f(R_{i}^{t})>f(R_{att})$$ (24)

For local optima, Remora selects a different method, as the next section demonstrates. If the attempted solution’s fitness function value is higher than the value of the current solution, the host gets to choose again.


$$f(R_{i}^{t})<f(R_{att})$$ (25)

*2*.*5*.*2*.*2 Eat thoughtfully (Exploitation)*. ***2*.*5*.*2*.*2*.*1 WOA strategy*.** The original WOA method was utilized to retrieve the position update formula of the Remora associated with the whale, as demonstrated by the following equations:

$$R_{i+1}=D\times e^{\alpha }\times \text{cos}(2\pi \alpha )+R_{i}$$ (26)

α=rand×(n−1)+1 (27)

$$n=-(1+\frac{t}{T})$$ (28)

$$D=|R_{best}-R_{i}|$$ (29)

Remoras on whales may be seen as having equal places in the greater solution space.

***2*.*5*.*2*.*2*.*2 Host feeding*.** Host feeding is another division of the invasion process. The geographic region of the host may now be the only viable option. Traveling on or around the host can be conceptualized as incremental steps, which have the following mathematical definition:

$$R_{i}^{t}=R_{i}^{t}+D$$ (30)

$$D=H\times (R_{i}^{t}-A\times R_{best})$$ (31)

H=2×S×rand−S (32)

$$S=2\times (1-\frac{t}{T})$$ (33)

Here, D was used to stand for a small movement related to the host and remora’s size space. To differentiate between the target and Remora, the location of Remora was limited using a Remora factor (A). The volume of the Remora is approximately 1% of the host’s volume if the host size is 1.

#### 2.5.3 Hybrid chaotic sand cat Optimization-Remora optimization

**Fig 4:**
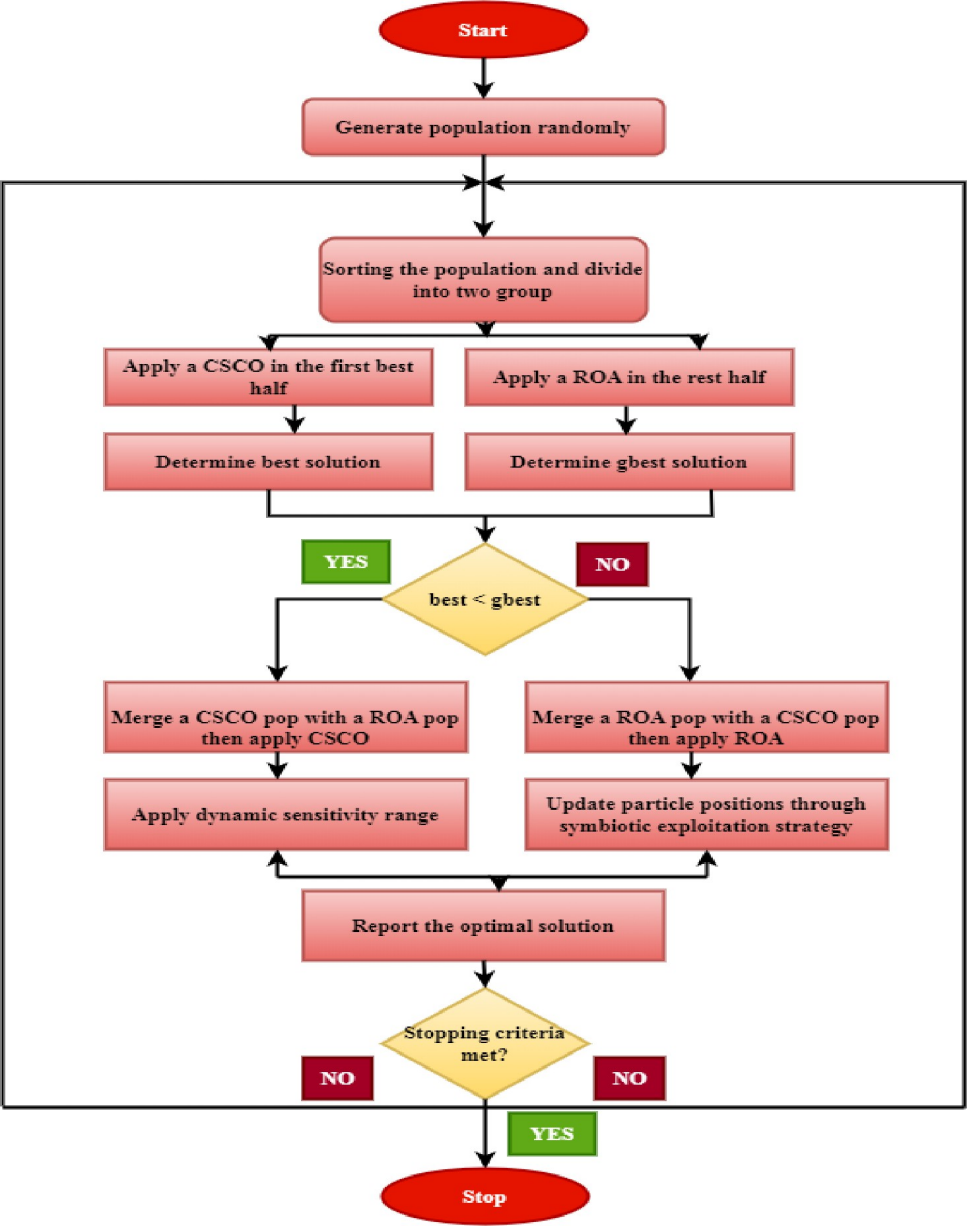
Hybrid feature selection algorithm of CSCO-ROA.

In solution search and optimization techniques, strong exploration and exploitation skills are needed. Finding the most intriguing prospective answers and conducting a comprehensive investigation of the search space are the objectives of the exploration phase. The goal of the extraction stage is to direct the search for the most practical answer for the people. A metaheuristic method’s accuracy and convergence speed can be increased by appropriately balancing the performance of exploration and exploitation. Combining the effective exploration and exploitation capabilities of the two algorithms aims to create a new algorithm that is even more powerful. To capitalize on the benefits and do away with the drawbacks of each technique, the two systems can be hybridized to blend their respective strengths into a single strategy. The suggested hybrid feature selection algorithm’s primary steps are displayed in below Fig 4.

### 2.6 Classification using proposed DL algorithm (CVAE)

Conventional learning methods are prone to low accuracy when handling data with several attributes. The drawbacks of conventional learning algorithms have been solved by our DL methods. We use two blocks in the VAE architecture an encoder block and a decoder block to understand the probability distribution of the information or A. The encoder maps A to a set of variables, *q*(*B*/*A*), which fully describe a set of related intermediate probability distributions. After sampling these intermediate distributions, a set of latent parameters, B, is created. This set of samples serves as the input for the decoder, the next block.

We must transfer the basic framework of the information to the probability distributions $q(B/A)\&p(\overset{\wedge }{A}/B)$ to achieve that goal. Since they represent the likelihood of B but rely on their particular inputs, B and A, respectively, $p(\overset{\wedge }{A}/B)$*q*(*B*/*A*) are the probability distributions and are conditional probability distributions.

**Fig 5:**
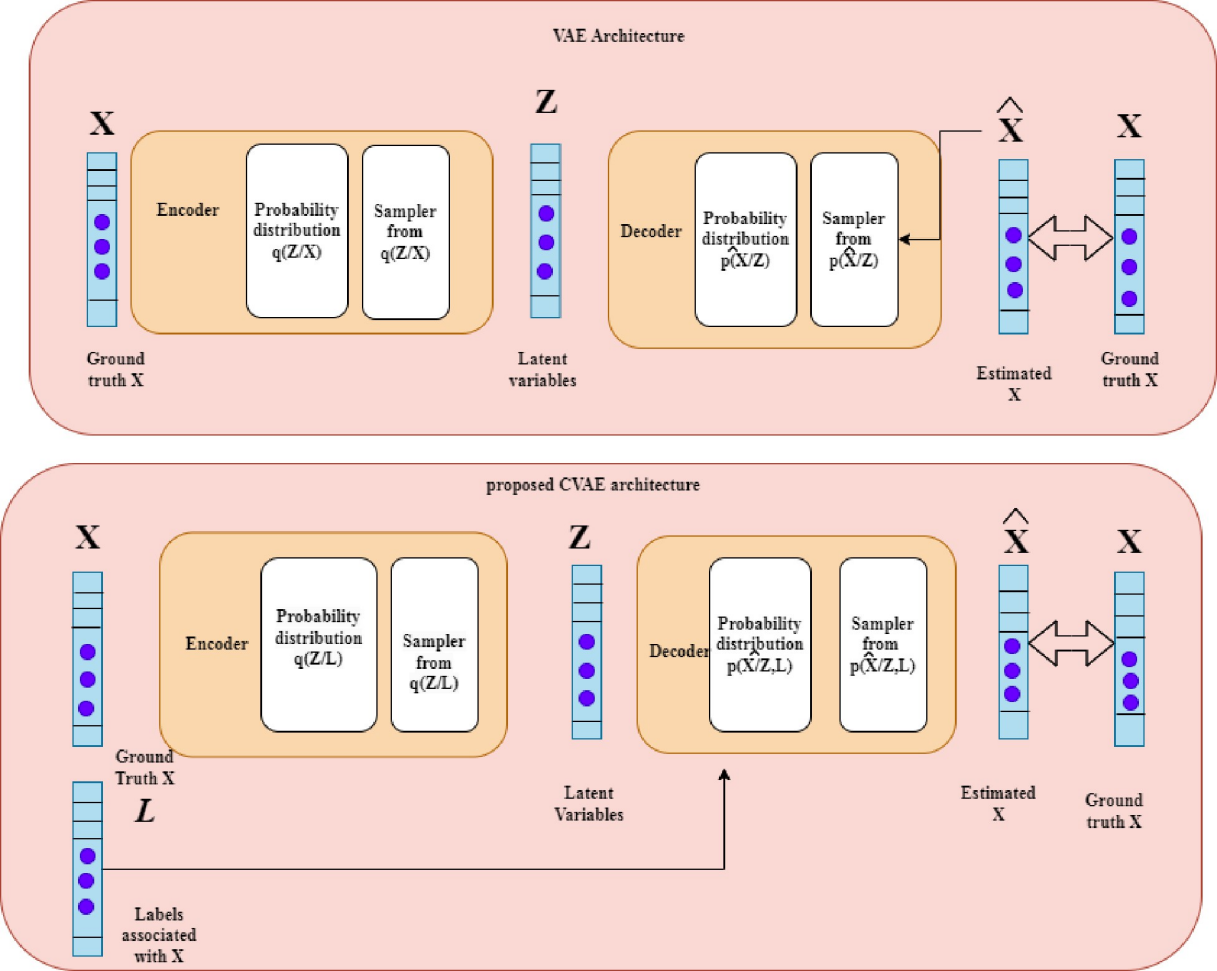
Comparing the proposed CVAE design to a standard VAE framework.

The variation technique, $q(B/A)\&p(\overset{\wedge }{A}/B)$ which simplifies the procedure of learning to a minimizing process and can be readily stated in terms of SGD in a neural network, is how we discover the probability distributions in a VAE. Fig 5 uses the model variables, *θ and φ*, to briefly depict the design. As part of the VAE training, these settings are adjusted.

By increasing a parameter known as the ELBO, we attempt to maximize the chance of receiving the necessary information as the outcome in the variation method.

Our proposed approach called CVAE, is based on the VAE model and is similar to a VAE in that it uses the classes of the instances as a further input to the second block rather than utilizing the same information for the network’s input and result. The encoder block remains unchanged when the labels are added as an additional input, but the decoder probability distributions are now conditioned on both the labels and the latent parameter. Incorporating the samples as additional input, while seemingly little, actually makes a big difference since it enables one to:

- Include additional data in the decoder block, which is necessary to establish the necessary coupling between the labels and the characteristic vector.
- Use all training data for categorization in a single training phase.
- Reconstruct features using this method. In the event of partial input instances, a proposed CVAE will acquire the pattern of characteristic values by them to the hidden distributions, from which a subsequent attribute recovery is carried out.

SGD may be used to minimize the function of loss for the CVAE model. As previously indicated, we can observe that the loss function consists of two components: a log-likelihood component and a KL divergence component. The second section considers the probability of generating A using the donation $p(\overset{\wedge }{A/B,L})$, or the space between A and $\overset{\wedge }{A}$. The distance between the range *q*(*B*/*A*) and a previous distribution for B may be used to understand the KL divergence portion. As a regularization phrase, *q*(*B*/*A*) we are truly preventing by minimizing this distance is deviating too far from its antecedent. This regularization term has the great characteristic of being automatically modified.

#### 2.6.1 Model details

**Fig 6:**
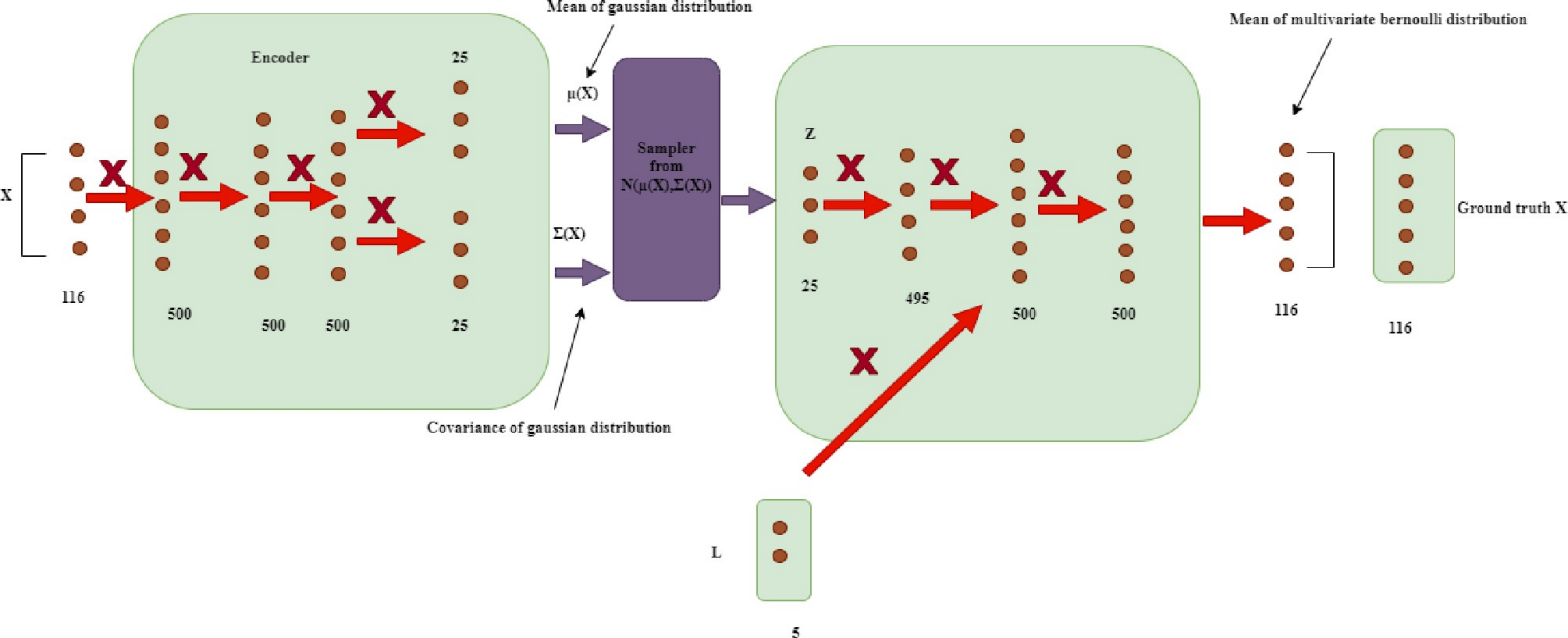
Proposed CVAE model.

The proposed CVAE model is detailed in Fig 6. As the distribution, we use a multivariate Gaussian *q*(*B*/*A*) with a vertical covariance matrix $\sum (A)\to \sigma_{i}^{2}(A)$, and a mean *μ* (*A*) that have distinct values along the diagonal. The previous distribution for Z is a regular normal *N (0*, *I)*.

In this instance, all we have to do is concatenate the label vector with the values from the decoder block’s second layer to obtain it within the decoder (Fig 6). The empirical results, after taking into account different places, have identified the location for putting the L labels. A completely linked layer is indicated in Fig 6 by a solid arrow with an adjacent X. Every layer has a number behind it that indicates how many nodes are in it. The final encoder layer’s linear activation function and the final decoder layer’s sigmoid activation function are the only two levels that have a different activation function than ReLU. The proposed approach uses CSCO-ROA to find the right hyperparameters to reduce the design parameters and achieve high efficiency, fast convergence, and low design parameters. Consequently, the proposed method shows great efficacy in raising classification accuracy.

## 3. Results

This part provides the findings of the recommended strategy for the classification of BrC through the use of graphs, confusion matrices, and numerical statistics. The updated, publicly available BreakHis histopathology datasets were used in the experimental process.

### 3.1 Dataset description

#### 3.1.1 BreakHis dataset

The BreakHis database was created in 2016 and is accessible over the internet. In total, there are 7,909 pathological images of clinical breast tumors collected from 82 patients. Of these, 2480 are benign tumors and 5429 are malignant tumors with magnifications ranging from 40× to 400×. The BreakHis database contains example images that are 700 x 460 pixels in size and are shown in RGB color style straight to the breast tumor pathology region.

### 3.2 Data augmentation

When a substantial quantity of training data is used, great accuracy and good performance are usually achieved. The reduced patient volume results in a limited number of samples in the biomedical databases. Thus, the use of data augmentation is essential. A technique known as "data augmentation" entails adding the amount of the input data by adding more information. There are several methods available for data augmentation. The present research employs rotation and flipping as approaches.

### 3.3 Evaluation metrics

The cross-validation approach was created to optimize the effectiveness, reliability, and validation of performance results across several datasets. Several criteria have been used to evaluate the detection efficacy of our suggested method. Some of them include the ROC curve, also called the AUC, sensitivity, F1 score, precision, accuracy, specificity, FDR, MCC, FPR, FNR, and NPV. The effectiveness of the recommended approach to current algorithms is evaluated using the previously specified factors as quantitative components. The following is a clear statement of the results of the measurement, which stand in for the present values being examined:

$$Accuracy=\;\left(\frac{\left(Tp+Tn\right)}{\left(Tp+Tn+Fp+Fn\right)}\right)$$ (34)

$$Precision=\;\left(\frac{Tp}{\left(Tp+Fp\right)}\right)$$ (35)

$$Recall=\;\left(\frac{Tp}{\left(Tp+Fn\right)}\right)$$ (36)

$$F1_{Score}=\;\left(\frac{2\;\left(Recall\;\times precision\right)}{Recall+precision}\right)$$ (37)

$$Specificity=\;\left(\frac{Tn}{\left(Tn+Fp\right)}\right)$$ (38)

$$NPV=\;\left(\frac{Tn}{\left(Tn+Fn\right)}\right)$$ (39)

$$FPR=\;\left(\frac{Fp}{\left(Fp+Tn\right)}\right)$$ (40)

$$FDR=\;\left(\frac{Fp}{\left(Fp+Tp\right)}\right)$$ (41)

$$FNR=\;\left(\frac{Fn}{\left(Fn+Tp\right)}\right)$$ (42)

$$MCC=\;\left(\frac{\left(\left(Tp\;\times Tn\right)-\left(Fp\;\times Fn\right)\right)}{\sqrt{\left(\left(Tp+Fp\right)\left(Tp+Fn\right)\left(Tn+Fp\right)\left(Tn+Fn\right)\right)}}\right)$$ (43)

### 3.4 Accuracy and loss graph

**Fig 7:**
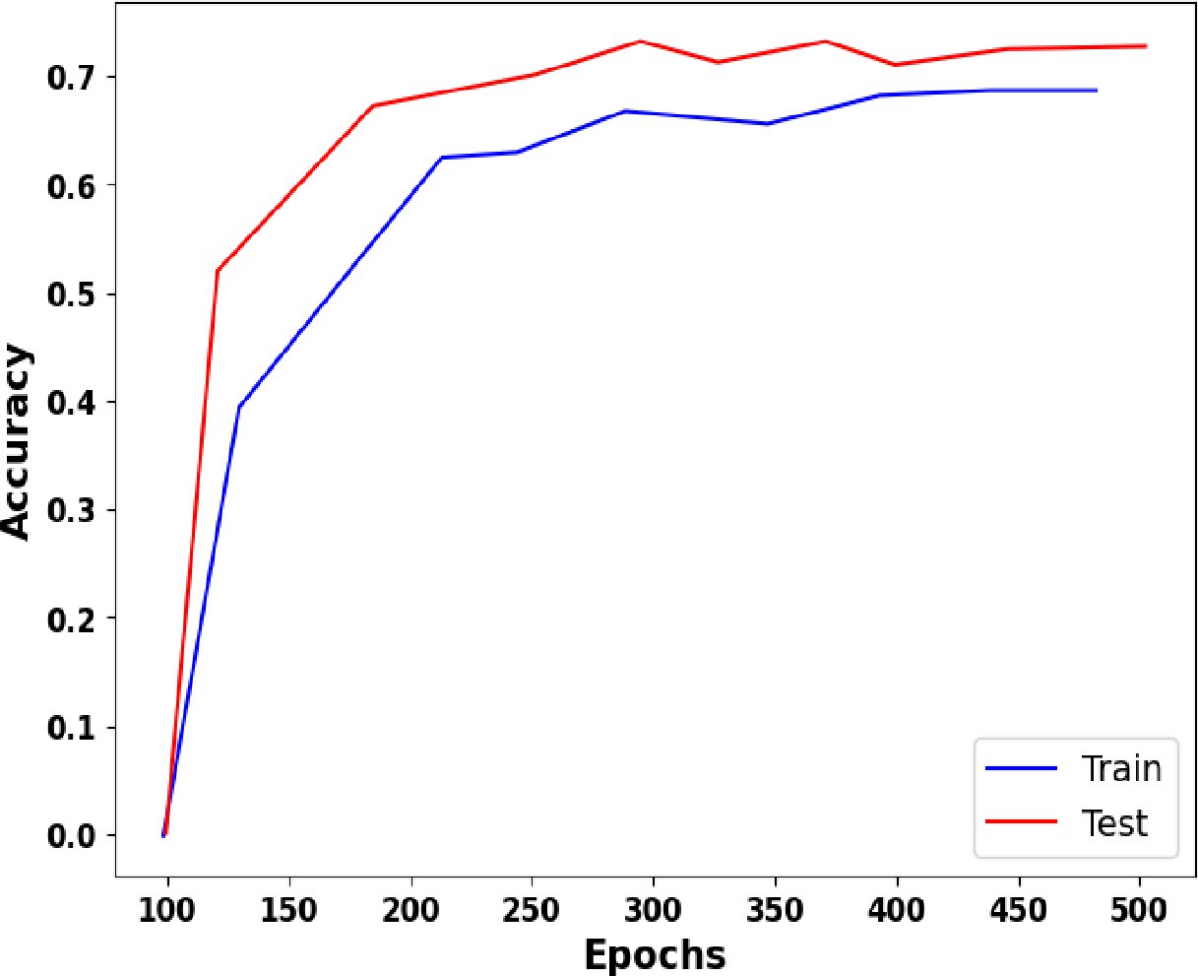
Accuracy vs. Epoch.

**Fig 8:**
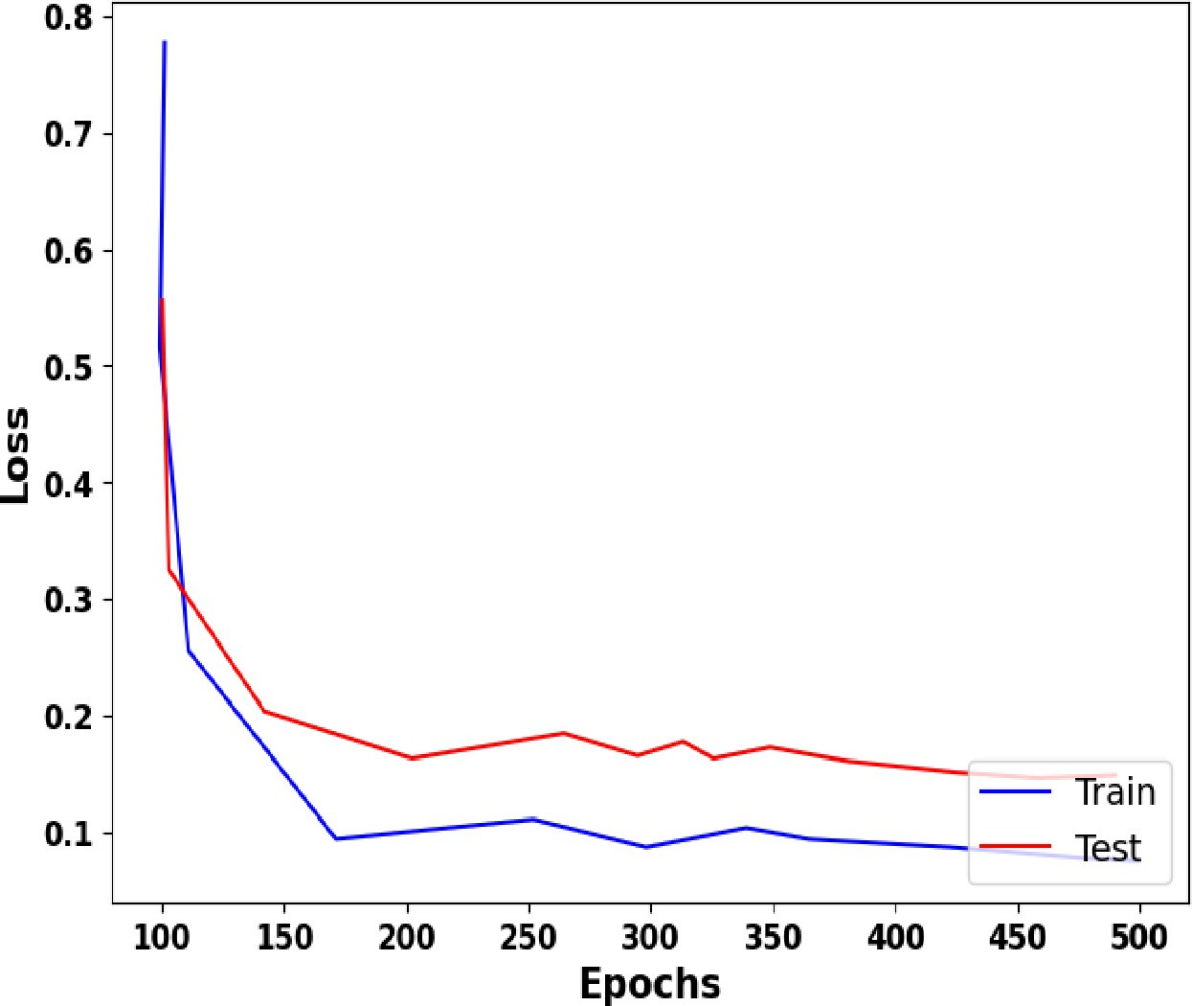
Loss vs. Epoch.

A graph illustrating the accuracy and loss results is presented in this section. The accuracy and loss graphs from the BreakHis dataset are shown in Figs 7 and 8. Test accuracy, test loss, training accuracy, and training accuracy were all considered in this study. Model classification efficacy is evaluated using accuracy and loss functions. The fact that the training and accuracy curves are quite comparable indicates that there isn’t an over-fitting issue.

### 3.5 5-fold cross-validation

Five-fold cross-validation testing was employed by the researchers to minimize bias error during diagnosis and confirm that every histological ROI in the collection of images was included in the test set only once. The Conditional Variation Autoencoder models are trained five times to get the overall effectiveness of the recommended categorization system.

### 3.6 Results on the BreakHis dataset

**Fig 9:**
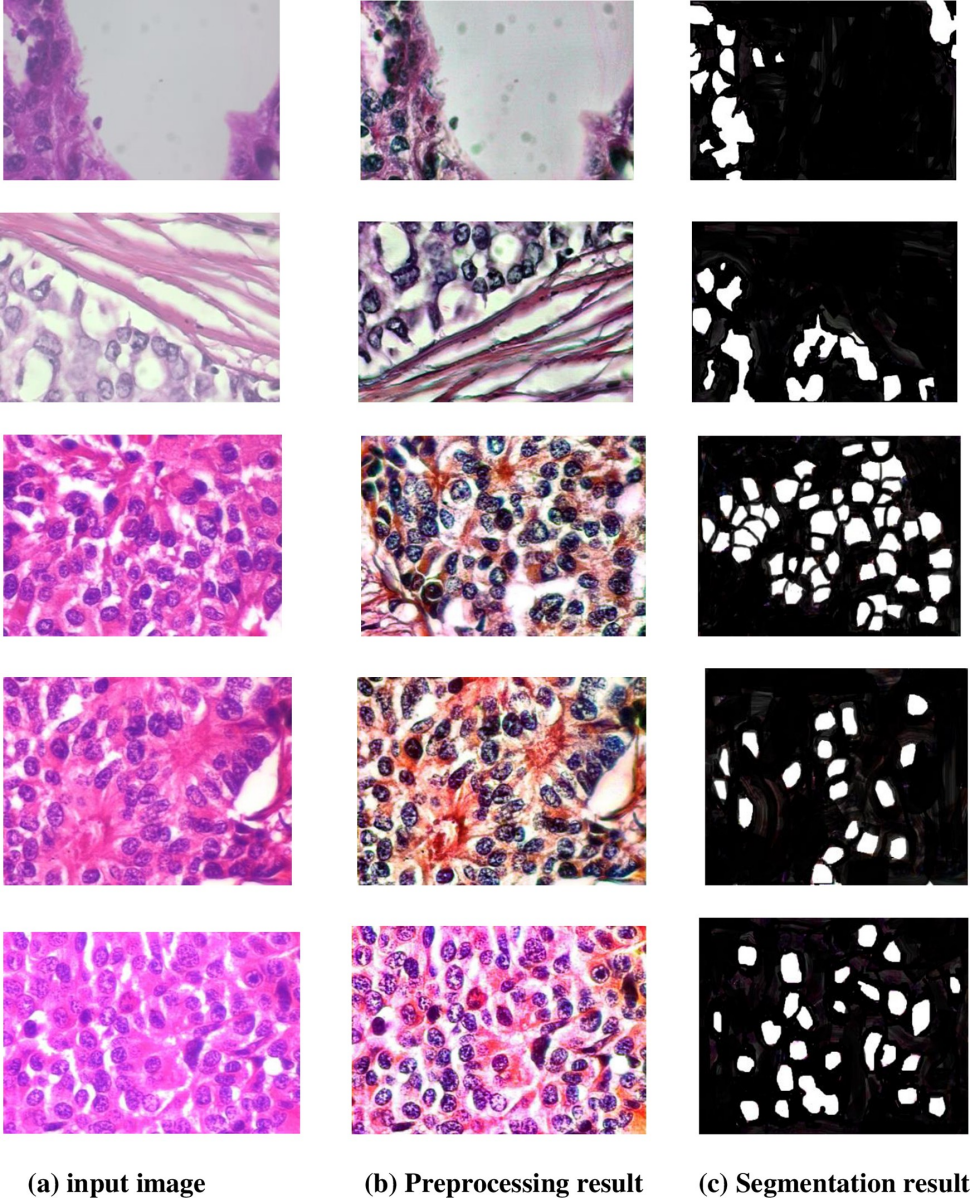
Segmentation results of the proposed model.

We provide the segmentation findings of the BreakHis dataset, which has been visually analyzed. Fig 9 displays the results of the BreakHis dataset’s breast mass segmentation process. Noise reduction, normalization, and enhancement have been applied to the preprocessed image, which is shown in the first column. As the ground truth zones that are important in the image, the genuine label maps are shown in the second column. To assess the segmentation models’ accuracy, these manually annotated masks are used as a reference. Regarding maintaining the margins of image masses of varying sizes and forms, the segmentation results of the recommended approach in the third column outperform those of other models. There is a strong similarity between the produced masks and the label maps of the ground truth.

The masks produced by the approach we propose are the most similar to the ground realities, indicating that they are better at distinguishing breast masses. The masks produced by the proposed algorithm show the greatest similarity between the segmentation results and the ground truth label mappings. Breast masses of any size or form can have their margins well preserved by the model.

### 3.7 Feature selection result analysis

Feature selection is a crucial step in DL, focused on identifying and extracting a subset of relevant features from a broad pool. This meticulous process aims to enhance model performance, address overfitting, and improve model interpretability. Our primary objective is to eliminate redundant and irrelevant features, optimizing the efficiency of our proposed model. In this research, a hybrid Chaotic Sand Cat Optimization-Remora optimization model is employed for feature selection. Initially, we used three feature sets—geometric, directional, and intensity to create a total of 33 features that describe the properties of the cell nuclei in each image. The below Table 1 succinctly outlines the features selected by each algorithm and the hybrid model for various feature types, facilitating a streamlined analysis of the feature selection results.

**Table 1:** Features selected by optimization algorithms.

Feature Type	Total features	CSCO selected features	ROA selected features	Hybrid (CSCO-ROA) Selected Features
Geometric	11	5	6	4
Directional	8	4	5	3
Intensity-based	14	7	6	5
Total selected	33	16	17	12

**Geometric features:** Computed concerning factors like perimeter, roundness, area, and number of fragmented cells.

**Directional features:** Capture irregular shapes associated with malignant and benign cells by calculating spatial distances in eight directions: West (W), East (E), South (S), North (N), Northeast (NE), Northwest (NW), Southeast (SE) and Southwest (SW).

**Intensity-based features:** Provide details related to mitotic cells, including standard deviation, mean, and range measurements.

Among the 33 features considered, the Chaotic Sand Cat optimization algorithm extracts 16, while the Remora optimization algorithm extracts 17. Our hybrid model evaluates the efficacy of these computed features in distinguishing between benign and malignant cases. The features are meticulously organized by our proposed hybrid model based on their effectiveness, revealing a notable drop in significance between the initial two features and the subsequent ones. Notably, the mean intensity value of the intensity-based set emerges as the second most impactful feature. In a broader perspective, directional features exhibit greater significance compared to geometrical and intensity-based features. Within our hybrid model, 12 features are employed for an efficient feature selection process. Despite some overlapping, the directional and intensity features play a pivotal role in differentiating the two classes. All 33 features are inputted into the classifier, reflecting a comprehensive approach that optimizes feature selection, thereby enhancing the overall efficiency and effectiveness of the model.

### 3.8 Confusion matrix analysis

**Fig 10:**
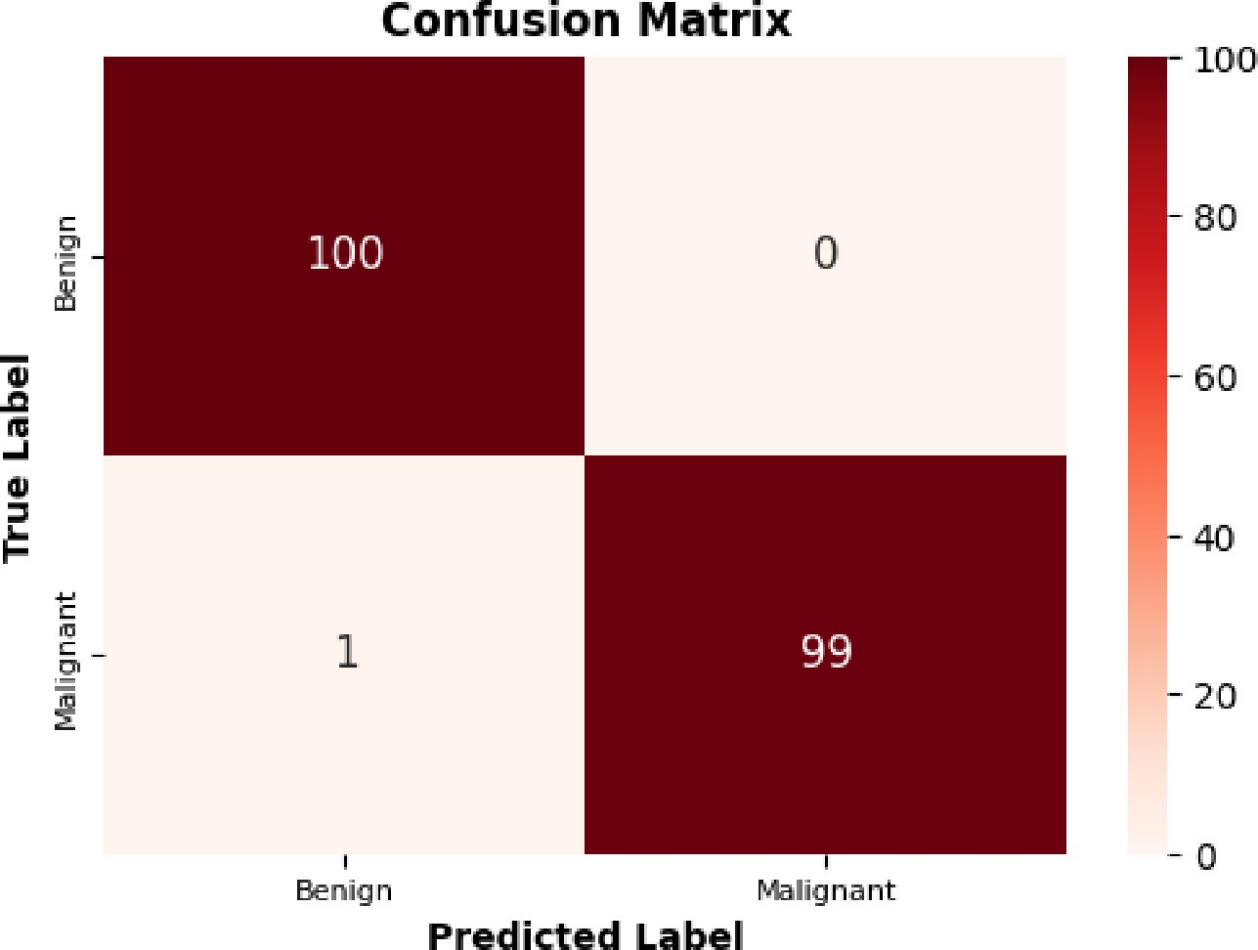
Confusion matrix for BrC classification on BreakHis dataset.

To assess categorization performance, a confusion matrix must be created initially. The generated confusion matrix in Fig 10 shows the applied test images. The model’s performance is shown by the confusion matrix used to categorize breast cancer as either malignant or benign. It demonstrates how the model accurately identified benign instances and incorrectly identified malignant ones, matching the real results.

The proposed model generated 1 false negative, incorrectly identifying malignant cases as benign, and 0 false positives, misclassifying benign patients as malignant. The algorithm forecasts benign and malignant instances with accuracy.

### 3.9 Performance of the classifier without and with feature selection

**Fig 11:**
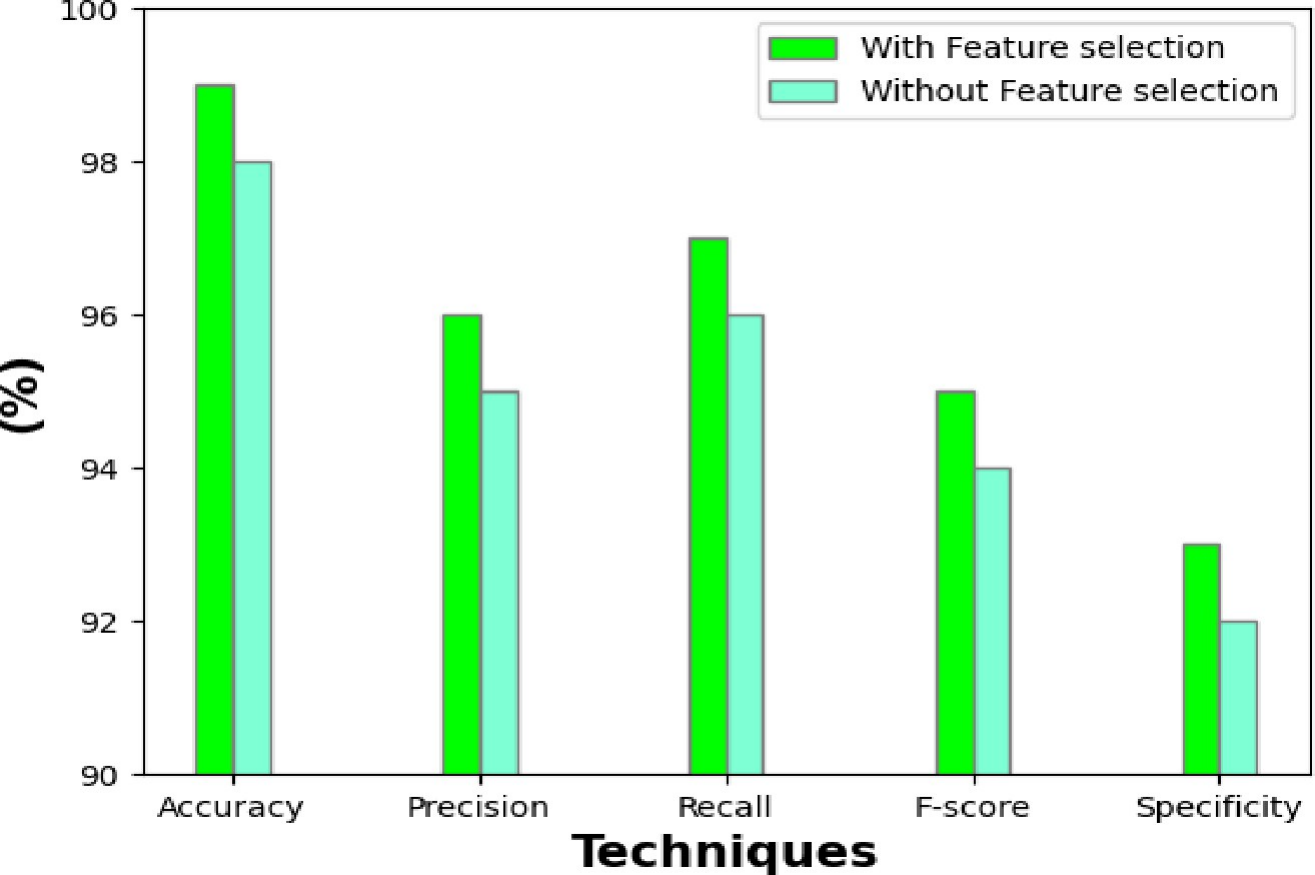
Performance of classification model with and without feature selection.

The performance parameters of a BrC identification model with two approaches—With FS and Without FS—are shown in Fig 11. The model’s accuracy with feature selection was 99.4%, meaning that 99.4% of the predictions came true. 99.2% of the instances that were correctly indicated were correctly classified, according to the accuracy rate. With a 99.1% recall rate, 99.1% of the real positive cases were likely properly recognized. With an F-score of 99%, accuracy and recall were measured in a balanced manner. With a specificity of 99.1%, the identification of negative instances was done with great precision. The model’s accuracy of 96.5% without feature selection indicates that 96.5% of the predictions were correct.

With an accuracy of 96.2%, 96.2% of the positive instances that were anticipated were appropriately categorized. Recall was 97%, suggesting a little reduced capacity to identify positive cases in contrast to the "With Feature selection" method. With an F-score of 96.4%, the recall and accuracy were not as well balanced. With a specificity of 96%, there was a modest decrease in the accuracy of identifying negative instances. These show that the technique "With Feature selection" fared better than the technique "Without Feature selection." The accuracy of breast cancer categorization was increased by feature selection.

## 4. Discussion

### 4.1 5-Fold cross-validation analyses

5-fold cross-validation is a technique for evaluating a categorization model’s efficacy in a BrC context. The dataset is to be divided into five equal folds. Every fold is utilized as a test set in the analysis, and the training set is the result of combining the remaining five folds. The entire process is run five times, using a new test set for each fold. Table 2 displays the assessment criteria of a cross-validation technique-based BrC classification algorithm before feature selection. F-score, Specificity, Precision, Accuracy, and Recall are among the measurements. Table 1 represents the average overall folds and each fold, which goes from the first to the fifth.

**Table 2:** Performance of classification model using 5-fold cross-validation (before FS).

Fold Test	Accuracy	Precision	Recall	F score	Specificity
1st fold	96.7	97.1	98	95.9	97.1
2nd fold	95.6	95.5	95.1	96.9	95
3rd fold	97.5	96.5	94	97	95.4
4th fold	95.5	95.5	96.2	97.2	96.2
5th fold	97	97.6	95.5	96.5	97.4
Average	96.46	96.44	95.76	96.7	96.22

After analyzing the data, we found that the model performed rather well. With an accuracy of 96.46% on average, 96.46% of the model’s predictions came true. With an average accuracy of 96.44%, the model appears to have correctly predicted 96.44% of the positive observations. The model was able to properly identify 95.76% of the real positive instances, as demonstrated by the average recall of 95.76%. The average value of the F-score, an equal measure of recall and accuracy, was 96.7%. The model’s average specificity, or its ability to correctly identify negative situations, was 96.22%.

The cross-validation analysis’s findings are shown in Table 3, which also includes measures of performance for each fold. The model can produce accurate forecasts, as evidenced by its average accuracy of 99.46%. The accuracy values, which range from 98.6% to 99.5%, show how well the model can detect affirmative cases. Recall values indicate that the model has a strong ability to identify true positive instances, ranging from 99% to 99.5%. Precision and recall performance are balanced, as shown by the F-score ranges between 98.9% and 99.2%. Additionally, the specificity values between 99% and 99.4% indicate how well the model detects negative instances.

**Table 3:** Performance of classification model using 5-fold cross-validation (after FS).

Fold Test	Accuracy	Precision	Recall	F score	Specificity
1st fold	99.7	99.1	99	98.9	99.1
2nd fold	99.6	99.5	99.1	98.9	99
3rd fold	99.5	99.5	99	99	99
4th fold	99.5	99.5	99.2	99	99.2
5th fold	99	98.6	99.5	99.2	99.4
Average	99.46	99.24	99.16	99	99.14

After feature selection, the accuracy of our suggested technique was substantially higher (99.46%) than that of other methods (SVM: 89.1%, ResNet: 97.77%, CNN: 98.80%). This significant improvement shows that we have selected key characteristics and captured important data for precise BrC classification with our approach. Our methodology seldom misidentifies negative instances as positive, which lowers the needless biopsies and related dangers. This is demonstrated by the low false positive rate (FPR: 0.002). Clinical requirements to reduce false positives are met by this. Our technique performed quite well (99.14%), indicating its capacity for accurately recognizing healthy persons, in contrast to SVM, which suffers with specificity (88.38%). In order to ensure optimal treatment for patients and prevent missed diagnoses, this is essential.

Overall, these findings show how reliable and effective the BrC classification method is in correctly categorizing cases of breast cancer across various dataset folds using pertinent feature selection.

### 4.2 Comparison results

**Fig 12:**
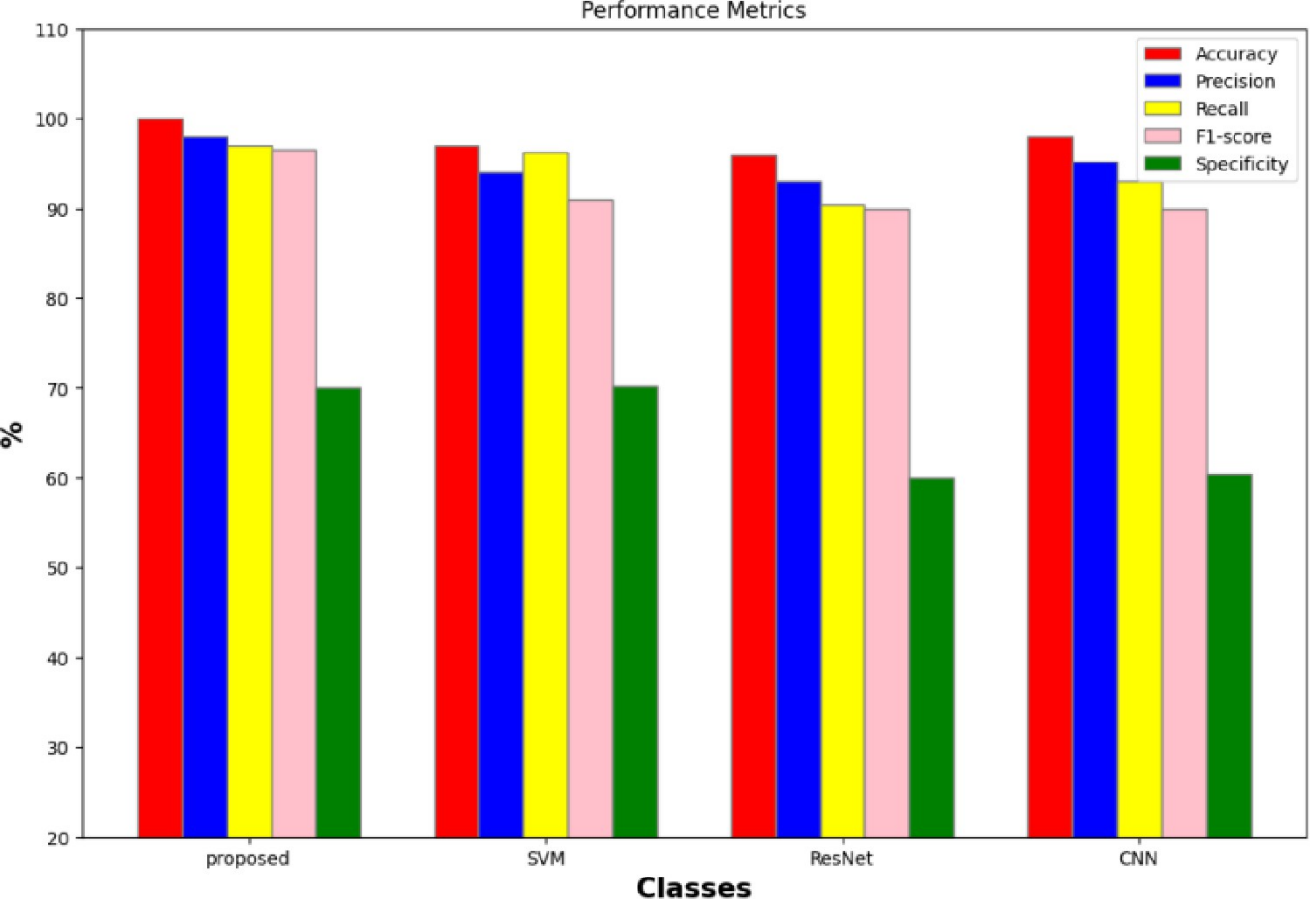
Performance of breast cancer classification technique.

The performance metrics of several methods, including the suggested method, SVM [42], ResNet [43], and CNN [44], are displayed in Fig 12.

Beginning with the proposed method, the greatest accuracy of 99.4% was attained. This shows that in 99.4% of the situations, the model’s predictions matched the actual labels. Additionally, it showed good recall (99.1%), pointing to a low number of false negatives, and excellent accuracy (99.2%), indicating a low rate of false positives. A performance that strikes a balance between recall and accuracy is indicated by an F-score of 99%. Furthermore, the proposed approach had 99.1% specificity, demonstrating its ability to accurately detect negative situations. Going on to the other methods, SVM demonstrated its effectiveness in reducing false positives and detecting positive occurrences with an accuracy of 89.1%, 86.67% sensitivity, 87.72% F-score, and 88.81% precision. However, its specificity was just 88.38%. ResNet attained a 97.77% accuracy rate. These findings imply that it is useful in categorizing instances of breast cancer. CNN’s results for BreakHis showed 98.80% accuracy, 98.84% precision, 98.35% sensitivity, and 98.59% F-score. These measurements show that it can detect affirmative instances while maintaining a good recall and accuracy ratio. To sum up, the proposed approach outperformed all other indicators in terms of accuracy and efficacy.

The outcomes for performance indicators related to a BrC classification model are shown in Table 4. The projected positive instances are FP, according to the FPR value of 0.003, which indicates a comparatively lower probability of inaccurate positive predictions. The model appears to be successful in properly recognizing the majority of positive cases, as indicated by the FNR value of 0.001, which shows a very low rate of false negatives. The low percentage of falsely identifying negative instances as positive is indicated by the FPR score of 0.002. This implies that the model can accurately detect negative situations with high precision. The model’s ability to accurately represent the real link between the characteristics and the categorization of BrC is demonstrated by the MCC value of 0.987, which shows a significant correlation between the predicted and real labels. Finally, the model’s efficacy in correctly categorizing the majority of negative situations is highlighted by the NPV value of 0.997, which indicates a high likelihood of effectively recognizing negative cases. The model appears to function well generally, as seen by the low FPR, the total correlation between predicted and real labels, and few FNs. The Table 5 shows the overall comparison of our proposed model with existing techniques based on the accuracy, dataset and FPR.

**Table 4:** Performance metrics for BrC.

Metrics	Results
FDR	0.54
FNR	0.001
FPR	0.002
MCC	0.987
NPV	0.997

**Table 5:** Compares the accuracy, dataset, and FPR of our suggested model with existing methods.

Method	Accuracy	FPR	Dataset
Proposed (After FS)	99.46%	0.002	BreakHis
Proposed (Before FS)	96.46%	0.009	BreakHis
SVM	89.10%	0.116	BreakHis
ResNet	97.77%	0.022	BreakHis
CNN	98.80%	0.012	BreakHis

In order to detect BrC, this paper presents a novel deep learning classifier called the Conditional Variation Autoencoder (CVA). When compared to previous approaches, CVA performs better because it efficiently learns global image patterns. In order to choose significant characteristics and reduce noise, the hybrid CSCO-ROA feature selection approach is unique and improves the accuracy and efficiency of the model. Our strategy outperforms the literature’s established approaches and provides outstanding performance across a wide range of criteria. This implies that it may be used in clinical settings and lead to better BrC diagnosis.

### 4.3 ROC analysis

**Fig 13:**
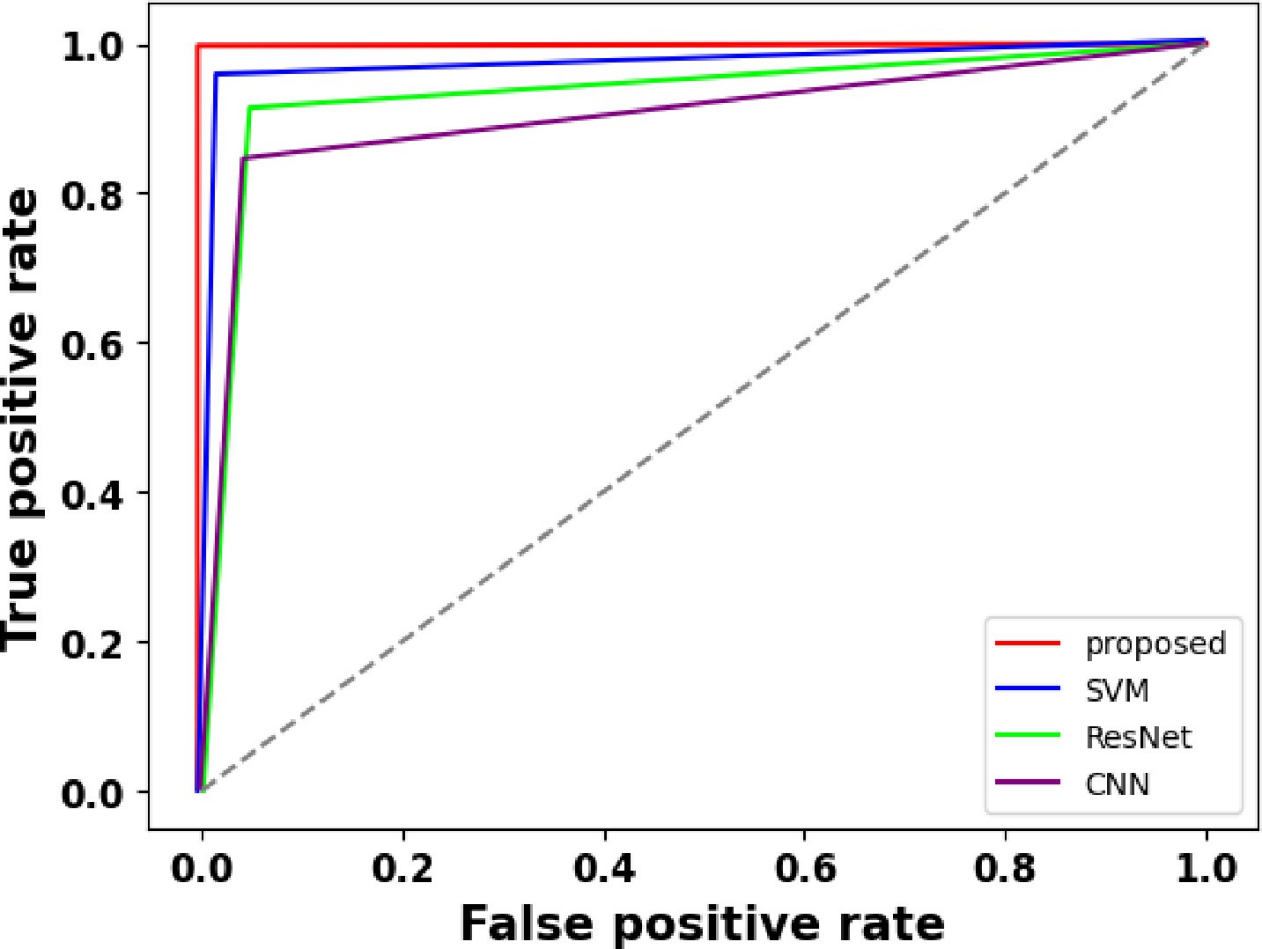
Comparison of AUC values for BrC classification techniques.

A ROC curve is a graphic depiction of a classification model’s performance at various categorization criteria. The TPR is shown against the complement of FPR in Fig 13. The AUC is a statistic used to assess a model’s performance. A higher AUC indicates better discrimination and classification accuracy. With an AUC of 0.99 in the provided data, the suggested method performed exceptionally well in differentiating between positive and negative cases of BrC. The AUC of the other three approaches was lower, showing a comparatively lesser capacity to discriminate.

The ROC with AUC values is a helpful tool for comparing and assessing the models’ performance. When assessing the effectiveness of BrC classification techniques, a high AUC is essential since it shows how well the model can differentiate between classes.

The approach we propose includes the Thresholding Level Set Method for segmentation and the ASMDBUTMF for pre-processing to eliminate noise. The attribute selection process employs a unique hybrid CSCO-ROA strategy, which significantly improves the model’s performance. Additionally, we create the Conditional Variation Autoencoder, a DL classifier for categorization that provides an accurate evaluation of the global structures present in images. Our experiments demonstrate that our model outperforms alternative testing strategies presently employed in the literature by a large margin. All things considered, our proposed method can significantly cut down on the time required to locate a BrC in a true emergency while still delivering respectable diagnostic results. Our research will aid in the creation of BrC categorization and segmentation techniques that are more precise and effective.

## 5. Conclusion

BrC affects a huge number of women worldwide, and if caught too late, it frequently results in death. When creating treatment strategies for their patients, clinicians would benefit immensely from an accurate categorization of BrC areas. In this work, we present a unique hybrid CSCO-ROA for feature selection to tackle this issue. To improve diagnostics and visualization, the research uses the ASMDBUTMF to remove noise from the input images. After that, segmentation is done using the Thresholding Based Level set approach. Following the segmentation phase, a hybrid feature selection approach is utilized to identify benign vs. malignant tumors using a DL classifier called Conditional Variation Autoencoder. We validate the proposed model’s efficacy for this histopathology image categorization work by conducting extensive experiments on the huge public BreakHis dataset. After assessing the model using various evaluation criteria, we report remarkable results from the proposed approach. Consequently, the classification accuracy reached an impressive 99.4%, accompanied by a precision of 99.2%, recall of 99.1%, F-score of 99%, specificity of 99.14%, FDR of 0.54, FNR of 0.001, FPR of 0.002, MCC of 0.98, and NPV of 0.99. These outcomes underscore the efficacy of our model in accurately categorizing histopathology images, providing clinicians with a powerful tool for diagnosing and distinguishing between benign and malignant tumors. While our findings contribute to the understanding of this research area, it is important to acknowledge certain limitations. Firstly, our proposed method demonstrates high performance on the BreakHis dataset; however, its effectiveness in diverse datasets or real-world scenarios remains unexplored, potentially limiting generalizability. The complexity introduced by the hybrid chaotic sand cat optimization technique and the Remora Optimization Algorithm (ROA) enhances feature selection but hinders interpretability and demands increased computational resources. Despite impressive classification metrics, there is a risk of overfitting the BreakHis dataset, posing challenges for generalization to unseen data. Addressing these limitations is crucial for refining and extending the applicability of our proposed approach in future studies. The proposed model is very sensitive and specific for a wide range of instances, which is useful for doctors and researchers who use pathological images to diagnose cancer. In the future, we want to evaluate the model’s effectiveness using a variety of datasets containing a range of cancer cases. We will concentrate on improving its efficiency in the future so that it can more precisely and instantly extract information from segment images.

## Supporting information

S1 Dataset (RAR)

## Abbreviations

BKD: Bilateral Knowledge Distillation
BrC: Breast Cancer
CAD: Computer-aided diagnosis
CapsNet: Capsule Network
CLSTM: Convolutional-LSTM
CNN: Convolutional Neural Network
CNNP: Non-Noisy Pixel Count
CSCO: Chaotic Sand Cat Optimization
CVA: Conditional Variation Autoencoder
DL: Deep learning
DNN: Deep Neural Network
ELBO: Evidence Lower Bound
FPR: False Positive Rate
GCN: Global Contrast Normalization
HPIs: Histopathology Images
KL: Kullback-Leibler
LSR: Label Smoothing Regularization
LSTM: Long Short-Term Memory
MWSA: Marker-Controlled Watershed Segmentation Algorithm
ROA: Remora Optimization Algorithm
SDC: Sequence Data Creation
SDRG: Stochastic Dilated Residual Ghost
SGD: Stochastic Gradient Descent
SVM: Support Vector Machine
